# The Canada-Guyana medical education partnership: using videoconferencing to supplement post-graduate medical education among internal medicine trainees

**Published:** 2017-04-20

**Authors:** William Stokes, Shannon Ruzycki, Ramdeo Jainarine, Debra Isaac, Joanna Cole

**Affiliations:** 1 University of Calgary, Alberta, Canada; 2 Georgetown Public Hospital Corporation, Guyana

## Abstract

**Background:**

A Guyana-based, internal medicine (IM) post-graduate medical education program was established in 2013. However, lack of formal teaching sessions are barriers to the program’s success.

**Objective:**

To describe the partnership between the University of Calgary and the University of Guyana’s internal medicine residency programs (IMRP). This partnership was created to support the Guyana’s IM academic half-day and is characterized by mutually beneficial, resident-led videoconference teaching sessions.

**Methods:**

Calgary medical residents volunteered to create and present weekly teaching presentations to Guyanese residents via videoconference. Questionnaires were completed by Guyanese residents and provided to Calgary residents as feedback on their teaching and presentation skills. A similar survey was completed by Calgary residents.

**Lessons learned:**

Twenty-four videoconference teaching sessions were conducted over eight months with a total of 191 and 16 surveys completed by Guyana and Calgary residents, respectively. Over 92% of both Guyana and Calgary residents agreed that the sessions enhanced their learning and over 93% reported increased interest in becoming more involved in international collaborations. 88% of Calgary residents felt the sessions improved their teaching skills.

**Conclusion:**

The formation of a resident-led, videoconference teaching series is a mutually beneficial partnership for Canadian and Guyanese medical residents and fosters international collaboration in medical education.

## Introduction

### Guyana

Guyana is a South American nation with a population of approximately 735,000 and a single accredited medical school in the capital city of Georgetown.^1^ Medical education in Guyana is five years of undergraduate education, followed by one year of rotating internship.

### Guyana’s brain drain problem

Brain drain is a critical problem in Guyana. Over 75% of individuals with a tertiary education permanently move abroad to live and work.^2^ Parallel trends in brain drain exists in Guyana’s health services, though the exact statistic is unknown. Brain drain within the health system is in part due to a lack of additional training opportunities in Guyana; prior to 2005, there were no postgraduate medical education programs in Guyana.^3^ Medical students wishing to pursue specialist training must travel out of country, usually Cuba or the United States (US), with many not returning to Guyana for work.^4^

Guyana lacks a sufficient number of physicians. For instance, Guyana has 2.1 physicians per 10,000 people compared to 2.1 physicians per 1,000 people in Canada.^1^,^5^ In the Georgetown Public Health Center (Georgetown, Guyana), a 400-bed tertiary care hospital, there are only three internal medicine (IM) physicians. Despite this, the IM service is one of the busiest in the hospital, admitting an average of 20 patients per day and seeing an average of 100–120 outpatients per clinic. For comparison, a 300 bed hospital in Calgary has seven IM physicians.^6^,^7^

### The Guyana internal medicine program

In 2013, the University of Guyana internal medicine residency program (IMRP) was created with the goal to improve physician retention and advance clinical care in internal medicine, with a focus on infectious diseases. Successful residents are conferred a Master’s degree from the University of Guyana at completion of the three-year program. At present, 14 residents are enrolled, with the first six residents having graduated in the spring of 2016.

The Guyana IMRP began with administrative support and leadership from the University of Maryland (Baltimore, Maryland) and funding from the US Centre of Disease Control (CDC). With completion of CDC funding in 2014, the IMRP has been financially independent from its American counterparts. New associations with the University of Calgary (Calgary, Alberta) and McMaster University (Hamilton, Ontario) aid the Guyanese IMRP in a variety of the program’s educational endeavours. Examples of such endeavours include weekly electrocardiogram (ECG) interpretation training with a Calgary-based cardiologist, weekly hematology rounds with the University of Pittsburgh, and intermittent didactic lectures from McMaster University, all of which are conducted via videoconferencing. In addition, Guyana residents are mandated to undergo a two month Canada-based elective.

### The Calgary-Guyana resident partnership

Delivery of an evidence-based internal medicine residency curriculum in Guyana has been hindered by a lack of qualified teachers. Our primary objective was to help fill this gap by having IM residents from the University of Calgary (U of C) establish a weekly, resident-led, teaching program using real-time videoconferencing. Our secondary objective was to increase knowledge and improve presentation and teaching skills of Calgary’s participating IM residents. Videoconferencing was used over other teaching modalities, such as online modules, recorded videos, or podcasts, for various reasons: its synchronous nature in which participants across sites could interact and engage with each other in real time; the availability of high quality videoconferencing equipment at both sites; and the mutually beneficial results previously demonstrated in similar international medical education partnerships using videoconferencing.^8^,^9^

The Guyana IM curriculum was divided into monthly modules categorized by IM subspecialties. Teaching topics are determined by the Guyana program director (author JC) and distributed to a U of C resident liaison (author WS) who recruits senior residents with specific interests in the topic area (see Table 1 for teaching schedule). The assigned Calgary resident created a one-hour, case-based, interactive presentation based on Royal College of Physicians and Surgeons of Canada learning objectives for internal medicine, with a particular focus on evidence-based medicine. At the request of the Guyana residents, an electronic copy of the presentation was distributed to them after each session.

Sessions were delivered weekly to the Guyanese residents in real time during their academic half-day via videoconferencing (between 1100–1400 Mountain Standard Time). The videoconferencing unit (PolycomR HDXR 8000) in Guyana was donated in 2014 through a generous grant from the Libin Cardiovascular Institute of Alberta. To minimize technical difficulties, administrative staff at both sites managed each videoconference setup, with information technologist support available as needed.

After each session, an anonymous questionnaire was completed by Guyanese residents and provided to Calgary IM residents. Results were provided to Calgary IM residents as feedback on their presentation content and organization as well as their teaching and presentation skills. The questionnaire used a Likert scale (1=very poor, 5=excellent) to assess the Guyanese residents’ opinions on the presentation’s organization, pace of delivery, involvement of participants, simplicity of material, and interaction. It also asked residents whether they increased their knowledge on the teaching topic, if the content was relevant, if they would recommend the session to other Guyana residents, if it made them more comfortable interacting with doctors from other countries, and if it motivated them to engage in international collaborations (1=strongly disagree, 5=strongly agree).

In addition, a similar anonymous questionnaire was completed by each Calgary resident teacher as feedback for the organizing committee on the overall experience (1=very poor or strongly disagree, 10=excellent or strongly agree). The five Calgary residents who were on the Guyana-Calgary partnership committee were excluded from completing the feedback survey. The questionnaire included questions asking whether the teaching was helpful for their learning, improved their teaching skills, improved their cultural awareness, increased their interest in presenting again, increased their interest in international collaborations, if they enjoyed interacting with the Guyana residents, made them more comfortable engaging with residents from other countries, and if they would recommend the teaching session to other U of C residents.

For comparison purposes, responses from Calgary residents were divided by two given the discrepancies in the 5-point and 10 point scales between the two surveys. Survey results were tabulated and means to graded responses analyzed using STATA, version 14.0.378 with associated 95% confidence intervals. The proportion of students who gave a positive response or agreed to each survey question, defined as ≥4/5 response, were reported.

After the completion of 17 teaching sessions, a short, twenty-minute focus group of the Guyana residents by the Calgary resident liaison (author WS) was conducted, in person, on February 26, 2016, in Georgetown, Guyana. This semi-structured focus group explored Guyana residents’ feedback, comments, and suggestions on the Guyana-Calgary partnership.

## What we learned

A total of 24 teaching sessions were conducted from September, 2015 to April, 2016 with a total of 191 and 16 surveys completed by Guyana and Calgary residents, respectively. Out of 14 Guyana residents, attendance averaged eight residents per session, with a range of five to 12 attendees.

Presentations from Calgary residents were very well received by Guyana residents. Guyana residents rated the presentations’ organization, pace of delivery, involvement of participants, interaction with the teacher, ease of understanding of materials, and ability to ask the presenter questions from very good to excellent with positive response rates ranging from 85 to 95% and means ranging from 4.31 to 4.48 (Table 2).

Guyana residents’ attitudes toward the presentations were also positive. Overall, 92% of Guyana residents agreed that sessions increased their knowledge on the topic discussed (mean 4.50), 96% agreed that the content of sessions were useful to their clinical practice (mean 4.55), and 95% would recommend the sessions to other Guyana doctors (mean 4.66) (Table 3). Furthermore, 94% agreed that the sessions made them more comfortable in interacting with Canadian residents (mean 4.63) and 93% wanted to become more involved in international collaborations (mean 4.63) (Table 3).

Feedback from Canadian residents revealed a positive experience. Overall, 88% of Calgary residents felt that participation in the session improved their teaching skills (mean 4.03), 94% felt it enhanced their learning (mean 4.03), 94% felt it increased their interest in international collaborations (mean 4.00), and 94% felt more comfortable interacting with medical residents from other countries (mean 4.00) (Table 4). Furthermore, all Calgary residents wanted to present again (mean 4.50) and all would recommend other Calgary residents to participate (mean 4.31). However only 63% of Calgary residents felt the teaching increased their cultural awareness (mean 3.47).

Ten Guyanese residents attended the focus group. They rated the audio and video quality as excellent. Guyana residents made several recommendations to improve the effectiveness of the presentations. They requested the presentation slides be distributed to them beforehand to help prepare for the lecture and suggested including more supplementary material to help with later studying. In addition, they asked for more in-depth discussion around the utility of laboratory investigations for diagnosis and management of disease, since in Guyana, access to laboratory tests is difficult and expensive. Guyana residents also expressed interested in presenting to Calgary residents.

## Discussion

### Other post-graduate medical educational partnerships in Guyana

Development of Guyana-based post-graduate medical education programs has been shown to be a crucial component in retaining Guyanese medical graduates. For instance, an eight-year partnership between the Canadian Association of General Surgeons (CAGS) and the University of Guyana to develop a sustainable, accredited post-graduate surgical training program was recently completed. The program graduated 14 Guyanese surgeons, of whom nine are practising in Guyana and five are completing additional training internationally.^3^,^10^ Similarly, a partnership between the Vanderbilt Department of Emergency Medicine and the University of Guyana established in 2010 led to the graduation of 11 accredited emergency physicians.^11^ In the Guyana IM program, the first set of Guyana IM residents graduated in spring 2016 with all six working in Guyana at the time of writing this paper. The Calgary-Guyana partnership’s goal is for the current graduates of the Guyana IMRP to continue working in Guyana and become involved in their IMRP academic half-day as teachers and organizers, such that the partnership will be replaced with effective, locally based teaching.

### Videoconferencing

Videoconferencing is a well-established, effective means of providing medical education. In a systematic review of studies, including four randomized controlled trials, that compared videoconferencing to face-to-face teaching of healthcare professionals there was no difference in teaching outcomes such as exam marks, differences in pre and posttests, and residents’ satisfaction.^12^ Within Canada, videoconferencing has also been used as an effective means in providing post-graduate medical education for distant sites.^13^–^15^ In Africa, videoconferencing among distant sites was also effective in providing medical education, though connectivity problems posed a much greater challenge^16^–^18^ No data could be found for the use of videoconferencing in medical education in South America.

Using videoconferencing for medical education between developed and developing countries is a relatively new process, but several studies have been conducted with good results. For instance, a low-cost, distant education program in obstetrics and gynecology conducted by residents and faculty at the Massachusetts General Hospital (MGH, Boston) and the Mbarara Regional Referral Hospital (MRRH) in Uganda was determined to be an effective and mutually beneficial educational tool, though no hard outcomes were measured.^8^

Furthermore, a trial comparing anesthesia teaching sessions that were teleconferenced from Harvard Medical School to Mbarara, Uganda demonstrated positive results for the Ugandan residents and the American residents who attended the sessions in person.^9^ In their study, both groups had statistically significant improvements in their pre-post lecture scores from attending 24 lectures conducted over one year. However, differences in pre-post test scores between the two groups were not examined.

In our study, both Guyana and Calgary residents felt the partnership contributed positively to their postgraduate medical education. Notably, Guyana residents felt that the videoconference lectures increased their knowledge on the topics discussed while Calgary residents felt the sessions improved their teaching skills. Both groups also felt that the sessions increased their interest in international collaboration. While these outcomes are subjective, they provide some evidence to support the use of international post-graduate medical education partnerships, through videoconferencing, in enhancing the education of both parties. Furthermore, it encourages and fosters a unique relationship between residents from first and third world health systems.

Limitations of this project include its inability to track objective outcomes such as effect of teaching sessions on examination scores or on clinical performance. Furthermore, the project mostly relied on end-user satisfaction with the partnership.

Many authors who examined videoconferencing in developing countries cite technical difficulties as the biggest limitation.^8^,^9^,^16^–^18^ This was a minor issue in our case; technical problems occurred infrequently, with three episodes delayed and one episode cancelled. The majority of videoconferencing issues occurred early on in the partnership. The lead author (WS) and a U of C IM administrative staff witnessed sessions from both countries and found the video and audio quality to be excellent and comparable between sites.

Difficulties were minimized by having information technologists available at each site.

Now past its first year, the Canada-Guyana IM teaching partnership continues with weekly videoconferencing sessions intended to supplement the Guyana IMRP academic half-day. One future direction for the Calgary-Guyana partnership is to expand lectures in various areas: skill building, such as sessions on residents as teachers; giving effective feedback; and creating teaching presentations. Furthermore, we are working to reciprocate our partnership by arranging for Guyana residents to present, via videoconference, to Calgary’s IM residents at their academic half-day.

The U of C and Guyana’s internal medicine program is an effective, resident-led, teaching program that enriches both parties’ post-graduate medical education and motivates residents to engage in international collaborations. With the improvement in quality, reduced operating costs, and accessibility of videoconferencing technology, international partnerships can become a valuable commodity for improving and enriching post-graduate medical education programs among participants.

## Figures and Tables

**Table 1:** Teaching topics from September, 2015 to April, 2016

Date	Teaching Topic
September 22, 2015	Nephrotic Syndrome
September 25	Nephritic Syndrome
October 16	Chronic Kidney Disease
October 23	Acute Kidney Injury
October 30	Hypertension
November 13	Myeloproliferative Disorders Bone Marrow Failure Syndromes Anemia
November 27	Breast Cancer Thombocytopenia
December 4	Multiple Myeloma Oncologic Emergencies
December 11	Gastrointestinal malignancies Lymphomas
February 5, 2016	Multiple Sclerosis
February 12	Peripheral neuropathies
February 19	Parkinson’s Disease
February 26	Dementia and Delirium
March 4	Depression and Suicide
March 11	Hyperkinetic Movement Disorders
March 18	Epilepsy
April 1	Anxiety
April 8	Intra-cranial Hemorrhage
April 15	Neuromuscular Junction Disorders

**Table 2:** Effectiveness of presentation delivery (N=175–191). 1=Very poor, 5=excellent

Objective	Proportion of students who gave a positive response (%)	Mean [95% CI]
Organization	180/191 (94)	4.47 [4.38 – 4.55]
Ease of understanding of material	181/191 (95)	4.45 [4.37 – 4.54]
Pace of delivery	168/191 (88)	4.36 [4.27 – 4.46]
Involvement of participants	163/191 (85)	4.31 [4.21 – 4.42]
Interaction with Canadian resident	159/175 (91)	4.42 [4.32 – 4.52]
Ability to ask the presenter questions	173/185 (94)	4.48 [4.40 – 4.58]

**Table 3:** Learner’s attitudes toward presentations (N=176 – 191]. 1= strongly disagree, 5= strongly agree

Objective	Proportion of students who agreed (%)	Mean [95% CI]
This session increased my knowledge on the topics discussed	176/191 (92)	4.50 [4.41 – 4.59]
The content of the teaching is useful to my clinical practice	180/188 (96)	4.55 [4.46 – 4.64]
I would recommend this session to other Guyana doctors like myself	176/185 (95)	4.66 [4.57 – 4.74]
This session makes me more comfortable in interacting with doctors from other countries	165/176 (94)	4.63 [4.54 – 4.72]
This session makes me want to be more involved in international collaborations	168/180 (93)	4.63 [4.54 – 4.72]

**Table 4:** Teacher’s attitudes on session (N=16). 1= strongly disagree, 5= strongly agree

Objective	Proportion of students who agreed (%)	Mean [95% CI]
This session was helpful for my learning	15/16 (94)	4.03 [3.70 – 4.36]
This session helped increase my teaching skills	14/16 (88)	4.00 [3.63 – 4.31]
I enjoyed interacting with the medical residents from Guyana	12/16 (75)	4.31 [3.95 – 4.67]
This session increased my cultural awareness	10/16 (63)	3.47 [2.91 – 4.02]
I would recommend other Calgary residents to participate	16/16 (100)	4.47 [4.19 – 4.75]
I would be interested in presenting to the Guyana residents again	16/16 (100)	4.50 [4.22 – 4.78]
This session makes me want to be more involved in international collaborations	15/16 (94)	4.22 [3.90 – 4.54]
This session makes me more comfortable interacting with medical residents from other countries	15/16 (94)	4.00 [3.38 – 4.62]
